# Schwannomas of the greater superficial petrosal nerve – case series, discussion of surgical techniques, and review of literature

**DOI:** 10.1186/s12883-022-02960-3

**Published:** 2022-12-09

**Authors:** A. Kaywan Aftahy, Maximilian Groll, Arthur Wagner, Melanie Barz, Denise Bernhardt, Stephanie E. Combs, Bernhard Meyer, Jens Gempt, Chiara Negwer

**Affiliations:** 1 School of Medicine, Klinikum Rechts Der Isar, Department of Neurosurgery, Technical University Munich, Munich, Germany; 2 School of Medicine, Klinikum Rechts Der Isar, Department of Radio-Oncology, Technical University Munich, Munich, Germany

**Keywords:** Greater superficial petrosal nerve, Middle cranial fossa, Neurosurgical oncology, Operative technique, Schwannoma, Skull base

## Abstract

**Background:**

Facial nerve schwannomas account for about 0.8% of all petrous mass lesions. Schwannomas of the greater superficial petrosal nerve (GSPN) are a rare subtype with few case-reports up to date.

**Case presentations:**

A retrospective analysis of clinical outcomes, radiographic findings and postoperative complication between June 2007 and December 2020 was performed. Four cases of GSPN schwannomas were reported. The presenting symptoms were facial nerve palsy and hearing loss. Imaging studies showed a subtemporal mass on the anterosuperior aspect of the petrous bone, in one case with extraordinary petrous bone and mastoid infiltration and destruction. Three cases were removed through a subtemporal extra- or intradural approach, one case via a combined pre- and retrosigmoid approach. Improvement of facial nerve palsy occurred in one case; new hearing loss was observed in another case. Xeropthalmia was a short-term temporary deficit in three cases. Short- to mid-term follow-up of the patients has not shown any tumor recurrence.

**Conclusions:**

GSPN schwannomas are rare entities presenting with heterogenous symptoms. Our surgical findings emphasize safe resection. Complete remission is possible by GTR. Since the small data set limits the expressiveness of statements regarding standard of care and alternative therapy options, additional data is needed.

## Background

Facial nerve schwannomas account for about 0.8% of all petrous mass lesions [1]. GSPN schwannomas are an rare subgroup, with just a few case reports up to date documenting different clinical and imaging characteristics of such lesions [2]. Differential diagnoses are meningiomas and trigeminal schwannomas, which can be distinguished in most cases by imaging modalities [3–5]. Middle cranial fossa schwannomas arise mainly from the trigeminal nerve originating from the petrous apex around the Gasserian ganglion and Meckel’s cave eroding the bone, whereas GSPN schwannomas tend to occur along its course in the mid portion of the petrous bone. There have also been reports of metastases spreading along the GSPN [4]. Surgical treatment remains a challenge. As it is crucial to identify the optimal treatment approach for this subgroup, we want to add our institutional and surgical experience of this rare entity and describe four successfully treated cases with satisfactory outcome followed by a review of literature in order to provide important diagnostic and treatment criteria for this entity.

## Material and methods

### Study design and outcome parameters:

An observational retrospective single-center case study series was performed. Patients who underwent surgery for GSPN schwannomas between June 2007 and December 2020 were included. The analysis of clinical records was performed considering the surgical approach, pre- and postoperative neurological status and adverse events during follow up visits. Extent of resection was determined by pre- and postoperative T1 ± contrast agent 3.0 T MRI sequences.

## Case presentations

### Case one

**Fig. 1:**
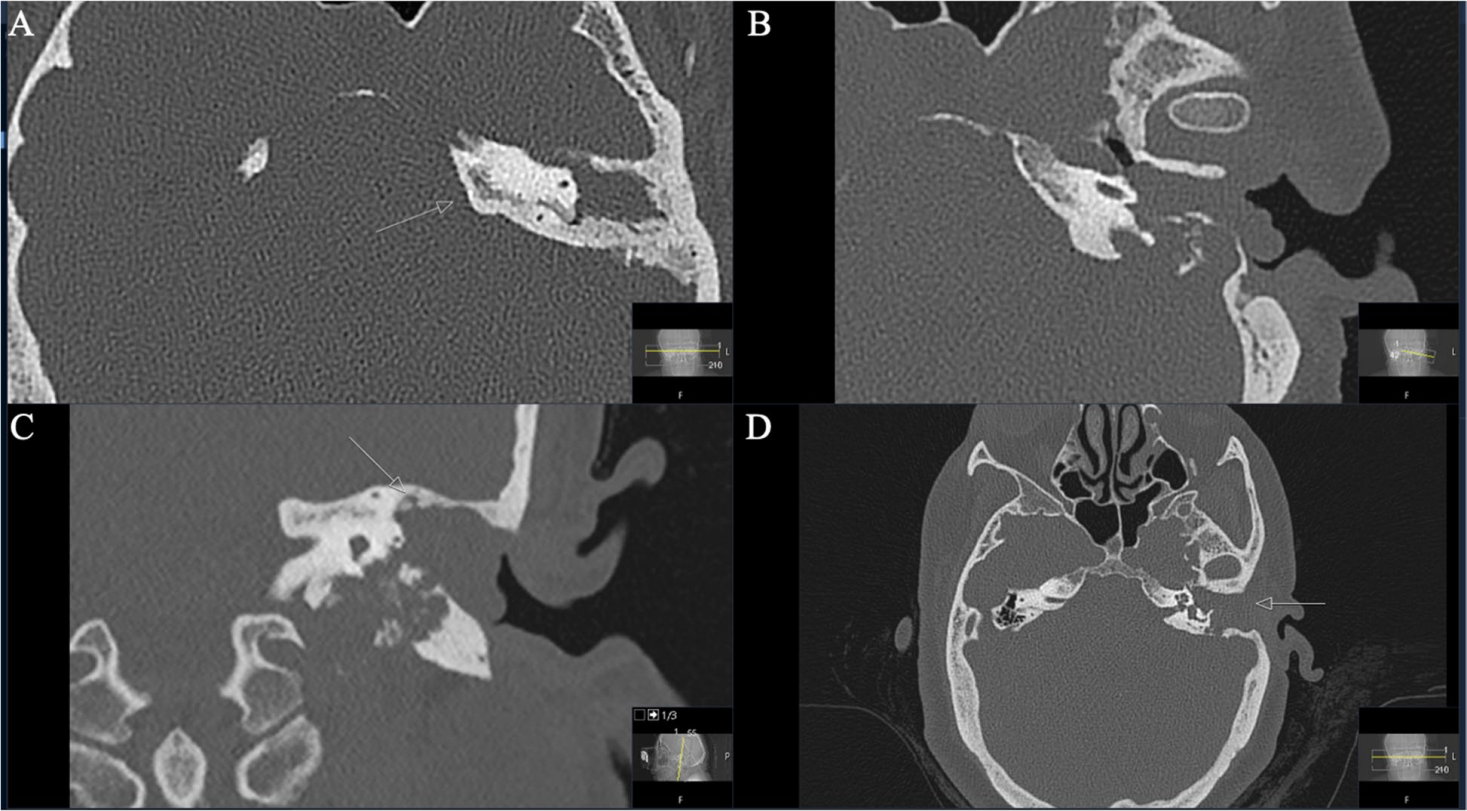
**A** Preoperative axial CT scan displaying an enlarged GSPN canal in the petrous bone (arrow) as well as osseous destruction of the mastoid. **B** A closer view showing no continuity of the auditory ossicles, with subtotal shading of the tympanic cavity. **C** Coronal reconstruction shows the tumor invading the petrous bone with osseous destruction. The tegmentum tympanii is thinned out (arrow). **D** Notice the mass effect in the mastoid (arrow)

**Fig. 2:**
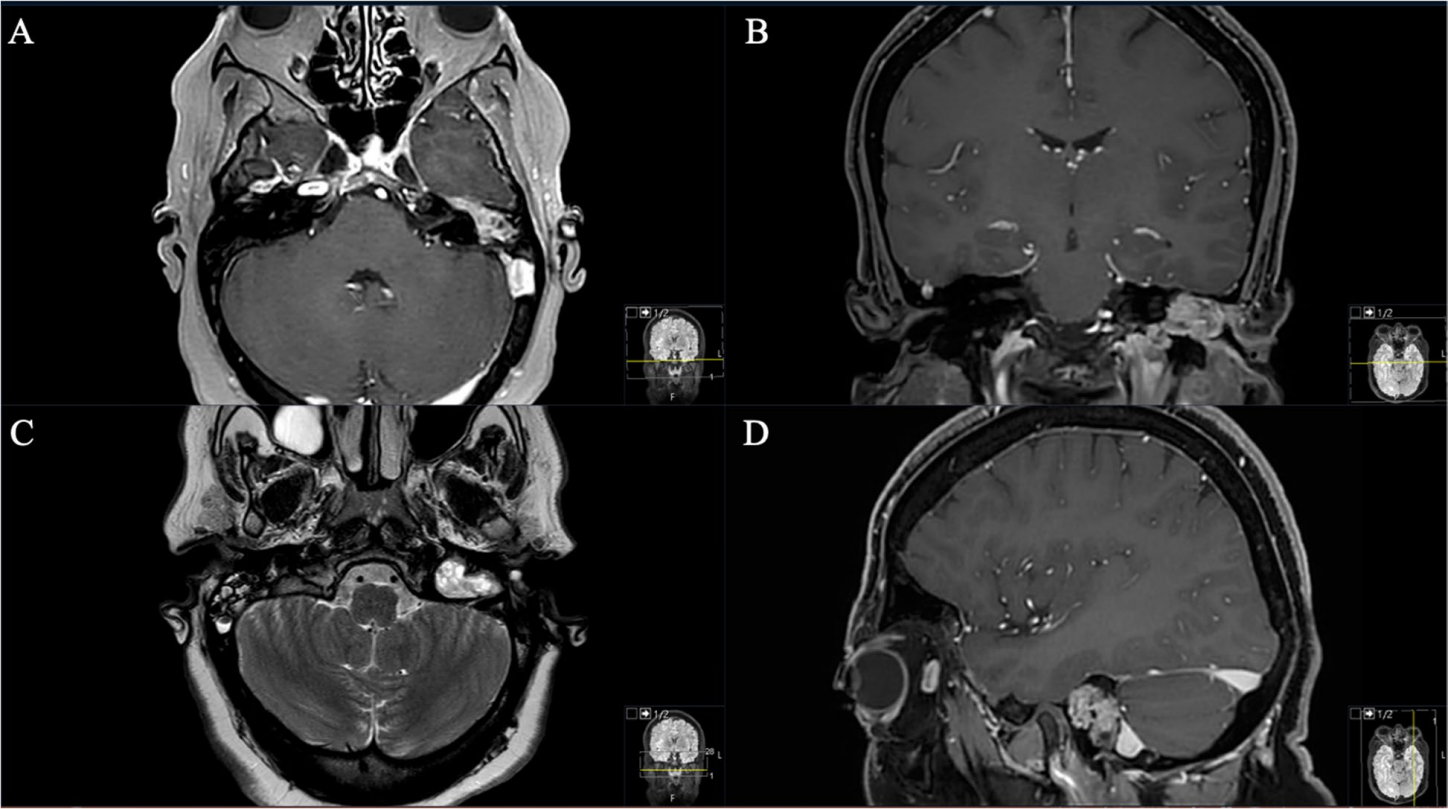
**A** Preoperative axial and (**B)** coronal T1-weighted gadolinium-enhanced MRI, showing the space-occupying schwannoma invading the petrous bone and the mastoid cells. A Notice the thickened and visible GSPN, the facial genu is not recognizable anymore. **C** Axial T2-weighted MRI sequence displaying the hyperintense homogeneous lesion in the petrous bone (**D**) Sagittal reconstruction showing the compressive effect on the sigmoid sinus (without any signs of a sinus vein thrombosis)

Reporting the first case of a 50-year-old woman presented with progressive facial nerve palsy. Presentation took place after a petrous bones mass on the left side was diagnosed by MRI. Biopsy of a schwannoma during a tympanum-exploration by our ENT department was performed. Regarding the patient’s previous history, it should be reported that a cholesteatoma had already been resected twice on the left side six years earlier. Both operations were performed by our ENT department. The patient reported a progressive facial nerve palsy on the left side (House and Brackman grade IV) for four weeks. She denied useful hearing. Audiometry revealed a pan tonal sensorineural hearing loss in the lower frequencies, on the left side combined with a conductive hearing loss up to 60 dB at 250 Hz (Figs. 1 and 2). MRI findings revealed a tumor mass with a volume of 5.63cm^3^.

### Surgical approach

An extradural subtemporal approach was performed in this case. A question mark-shaped incision was chosen and a temporal craniotomy was carried out with additional drilling to the skull base. The middle cranial fossa was exposed extradurally to the anterior portion of the petrous edge and the lateral edges of the oval and round foramina. A brain retractor was placed. Important landmarks for orientation during the extradural dissection are the middle meningeal artery (MMA) and the superficial greater petrosal nerve (GSPN). After coagulating and cutting the MMA, the GSPN was identified. The petrous bone seemed to be pathologically changed, drilling with a 3-mm diamond bit was performed to expose the facial knee. The petrous bone and also the mastoid cells have been fully infiltrated and destructed by the tumor, as expected. Intraoperative facial nerve monitoring remained stable, acoustic evoked potentials showed no remarkable decrease. The tumor, attached to the facial neve and the GSPN could be removed by careful dissection. A mastoidectomy was performed additionally to ensure GTR, doing this, a pre-existing fistula to the external auditory canal was recognized. The fistula was treated conservatively with regular follow ups by the ENT department.

### Histological findings

Parts of a moderately cell-rich spindle-cell tumor with an elongated, moderately chromatin-dense nucleus and a pale eosinophilic cytoplasmic border could be recognized. Immunohistochemically, the spindle-cell tumor showed a strong positive reaction against S-100. No reaction was found against GFAP, EMA, progesterone receptor, MAP-2 and pancyotkeratin. Ki67 proliferation index (MIB1) within the spindle cell areas was 3%. The final diagnosis was made after completion of 850 k methylation analysis with the result of a schwannoma, WHO-grade I.

### Surgical outcome and follow-up

**Fig. 3:**
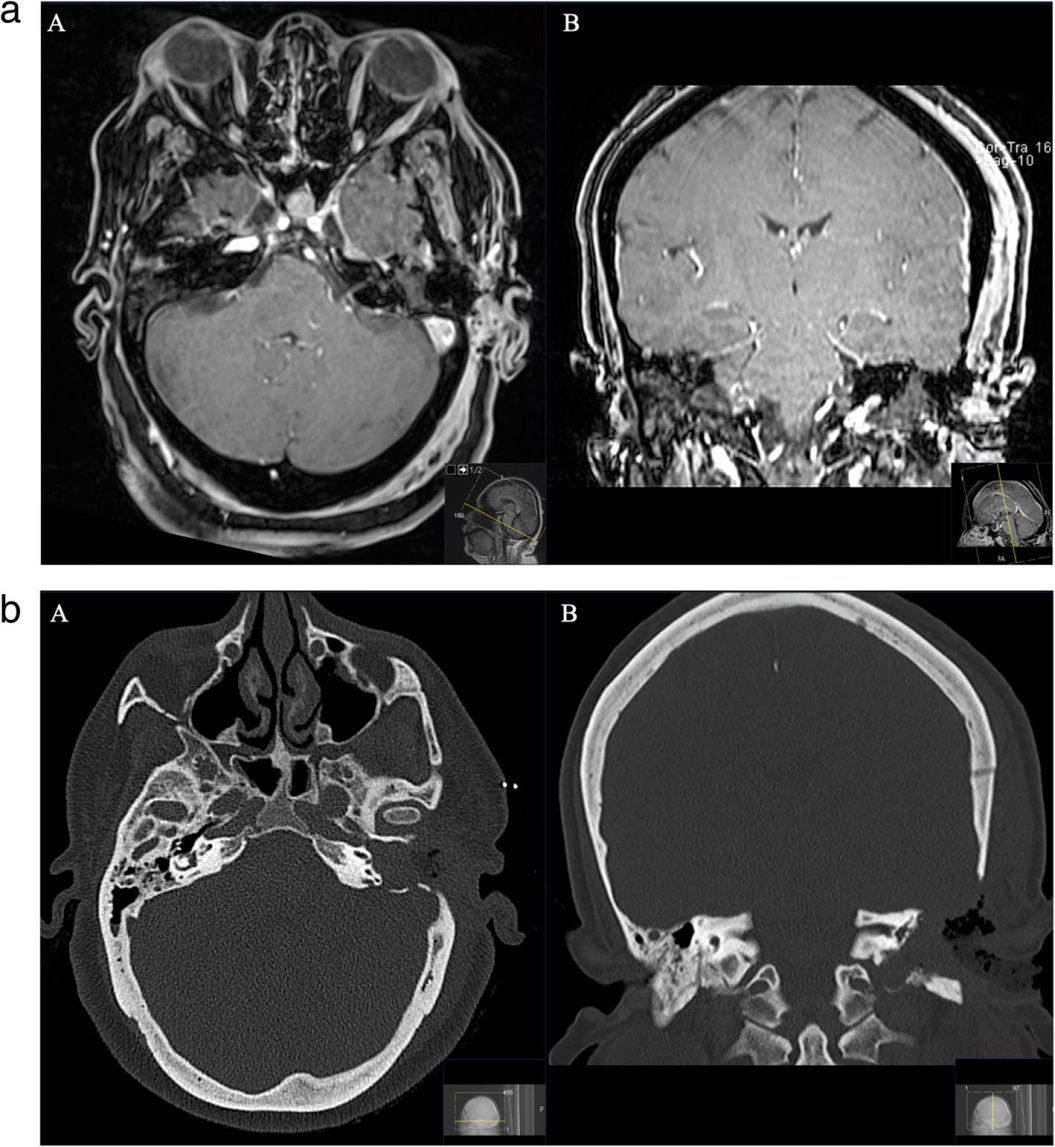
a: A Postoperative axial and B coronal T1-weighted gadolinium-enhanced MRI control, showing complete resection of the schwannoma. b: A Postoperative axial and B coronal CT scan, showing the extent of resection and the performed approach. Intraoperative findings of a complete infiltrated mastoid led to the surgical decision to opt for radical mastoidectomy

The postoperative course was uneventful without new focal neurological deficit. Postoperative MRI confirmed complete removal (Figs. 3a, b). Subsequent postoperative nausea and vertigo regressed completely during the inpatient stay. Outpatient controls have been performed regularly by ENT; a surgical revision of the fistula was not necessary. Due to the facial nerve palsy which remained unchanged after surgery plastic reconstructive surgeries were planned (Fig. 3a and b).

### Case two

**Fig. 4:**
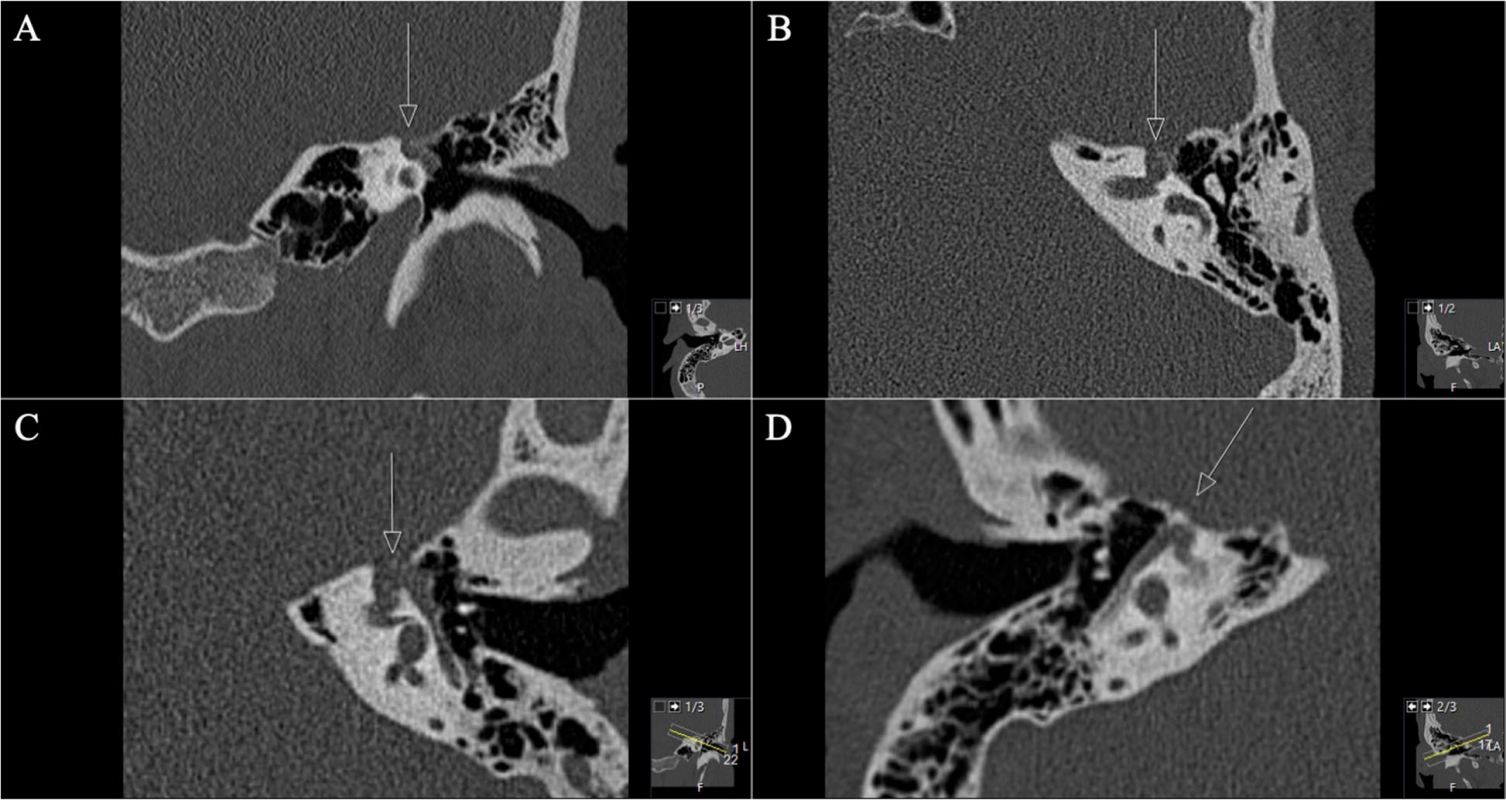
**A** Preoperative coronal and (**B)** axial CT scan, showing a widened facial knee and GSPN canal (arrows), the osseus tegmentum tympanii seems to be thinned out. In addition, notice the osseous discontinuity with the accompanying destruction of the canal roof of the petrous bone. Coronal comparison of the affected left (**C**) and the healthy right side (**D**) to highlight the anatomical differences (arrows)

**Fig. 5:**
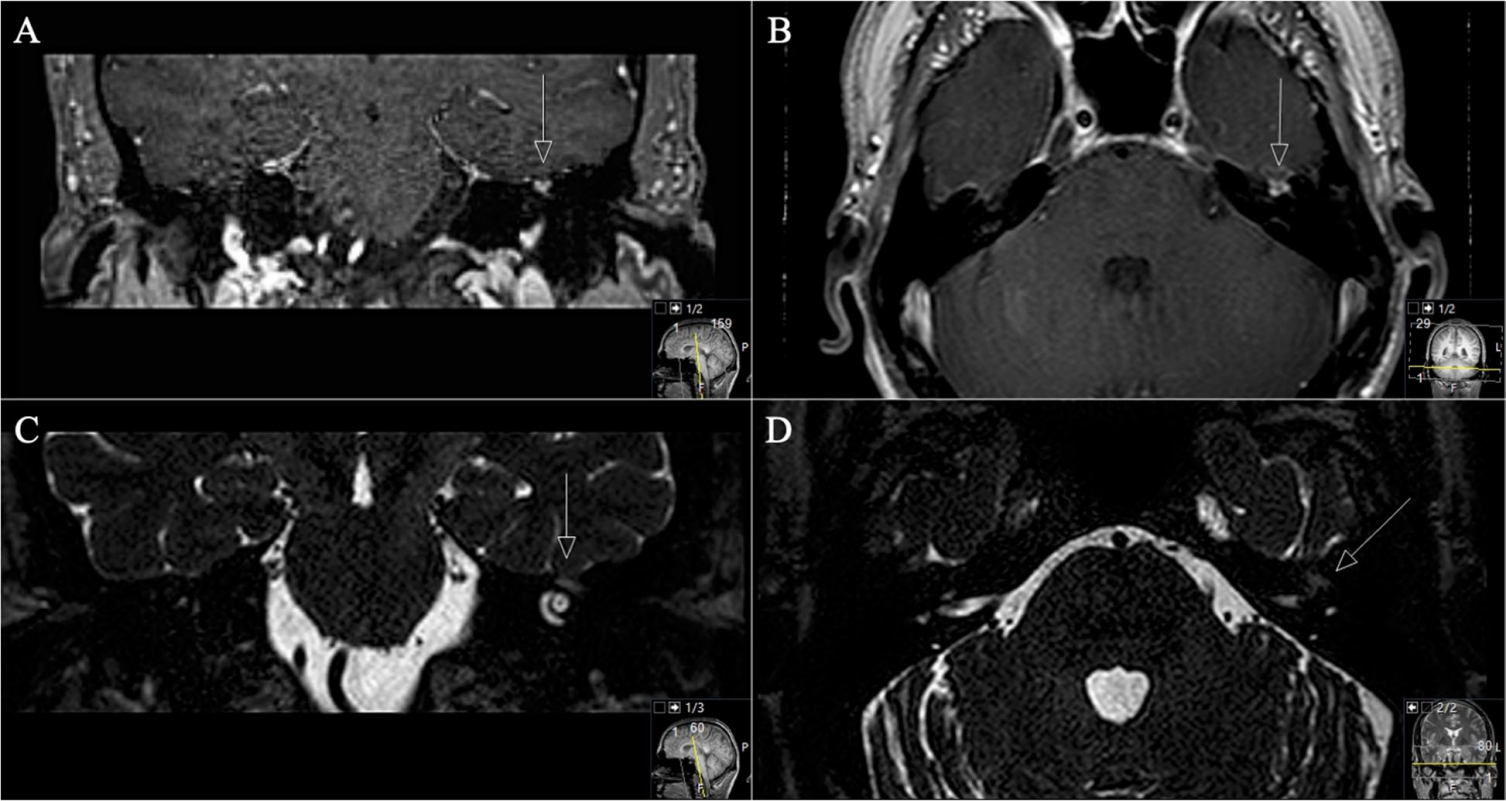
**A** Preoperative coronal and (**B**) axial T1-weighted gadolinium-enhanced MRI, showing a subtemporal small homogenous enhancing lesion in the middle cranial fossa on the petrous bone in the vicinity of the facial knee. **C** Coronal and (**D**) axial three-dimensional constructive interference in steady state (CISS) MRI sequences, highlighting the small hyperintense overlookable lesion next to the semicircular canals. Notice how the hypertense signal stops with the intraosseous entry of the canal at the fallopian hiatus

Reporting the second case of a 37-year-old man presented with progressive left facial nerve palsy (House & Brackman grade IV) for 2 months. ENT examination confirmed normal hearing. Electrophysiological examinations revealed axonal damage to the facial nerve with 50% reduction of amplitude (Figs. 4 and 5).

#### Surgical approach

An extradural subtemporal approach was performed in this case as well. Preoperatively, a lumbar drainage was placed and 30 ml of CSF released. A C-shaped horseshoe incision and a rhomboid temporal craniotomy with the squamous suture as the cranial border was performed, additional drilling was used to achieve a smooth osseus plane corridor to the middle cranial fossa. The middle cranial fossa was exposed extradurally to the anterior portion of the petrous edge and the lateral edges of the oval and round foramina. Due to sufficient CSF release no brain retractor was needed. After cutting the MMA, the GSPN was exposed. Dorsal to the GSPN, the petrous bone was destructed, intraoperative navigation confirmed the localization of the facial knee. Meticulous drilling with a 3-mm diamond drill was performed until the facial knee was fully exposed. Intraoperative facial nerve monitoring showed few spontaneous activities during this procedure, but acoustic evoked potentials remained stable. The tumor was exposed, carefully dissected and successfully removed (tumor volume 0.85cm^3^). The facial nerve and the GSPN showed no signs of injury and had normal thickness and appearance, the tumor was just attached to the nerves but also separated by a tumor capsule.

#### Histological findings

No immune reaction against epithelial membrane antigen could be found. The tissue did not show an S-100 immune reaction, a schwann-cell process was unlikely, but portions of a moderately cell-rich schwann-cell tumor was found. The diagnosis of a schwannoma, WHO-grade I was made interdisciplinary by neuro-oncological board discussion.

#### Surgical outcome and follow-up

**Fig. 6:**
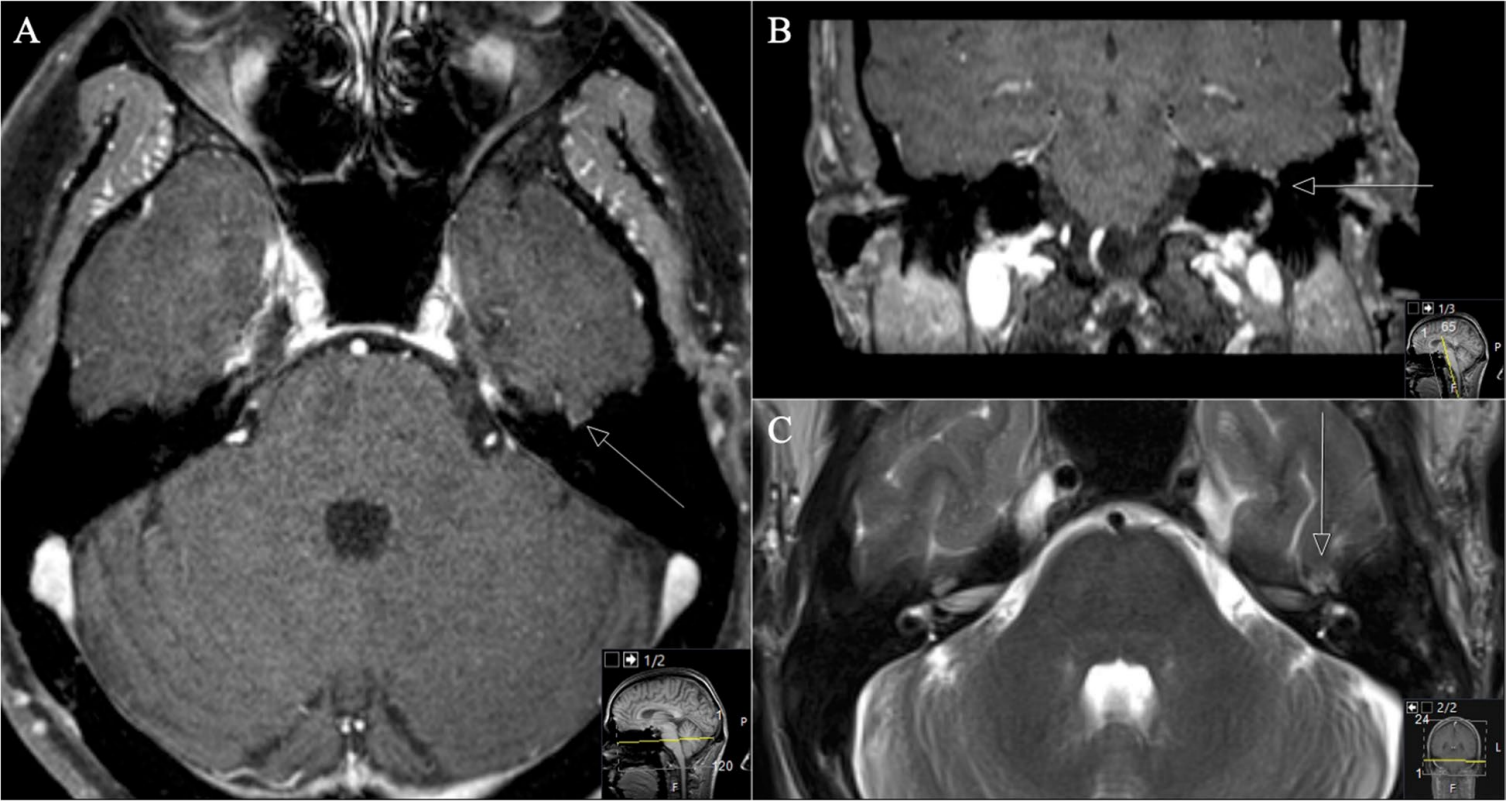
**A** Postoperative axial, (**B**) coronal T1-weighted gadolinium-enhanced and C T2-weighted MRI control indicating complete resection of the GSPN schwannoma (arrow)

The postoperative course was uneventful without any new neurological deficits (Fig. 6). The facial nerve palsy remained unchanged. The 3-month follow-up did not show any new symptoms or neurological deficits. There was a subjective discrete improvement of the facial palsy, as well as an almost complete eyelid closure. An increased lacrimal secretion on the left side occurred as a sign of recovery of the parasympathetic fibers. The 9-month follow-up showed no new symptoms as well. The facial nerve palsy improved to House and Brackman grade III (Fig. 6).

### Case three

**Fig. 7:**
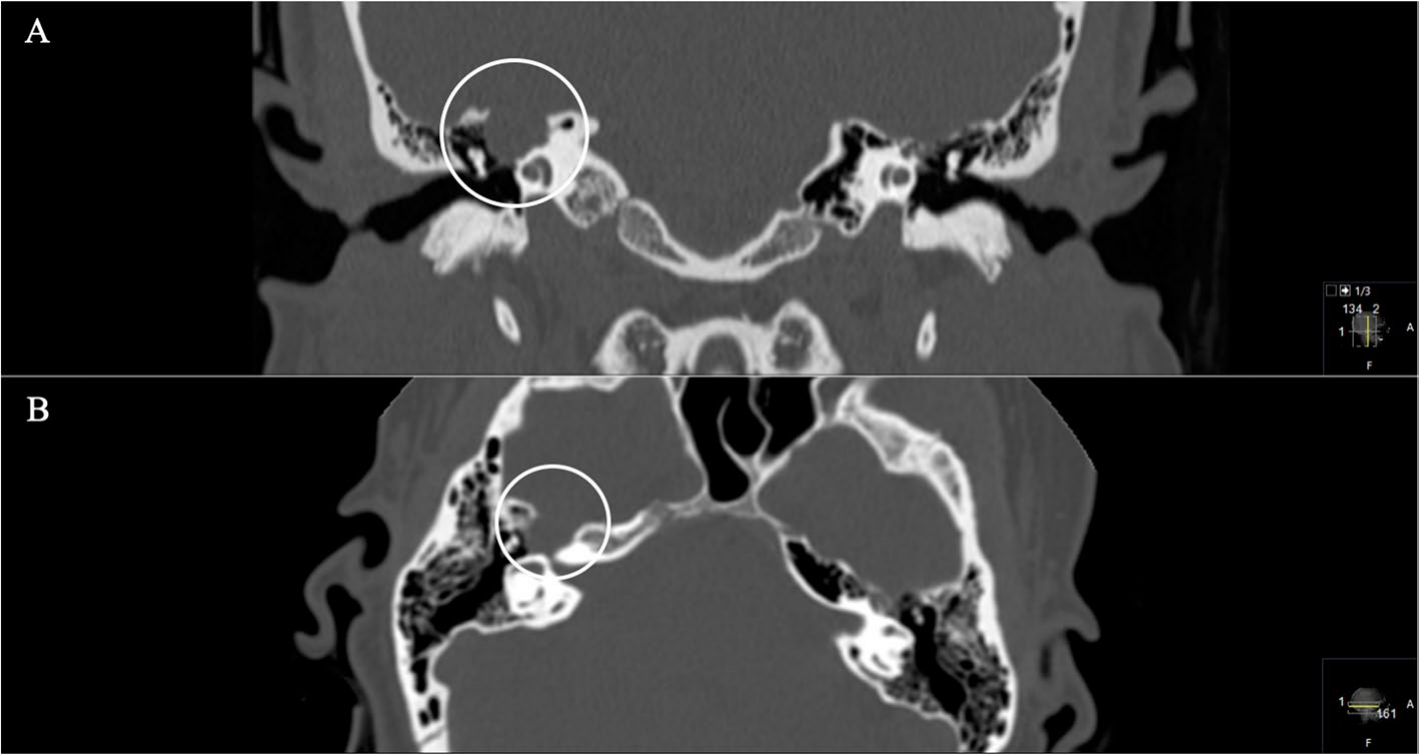
**A** Preoperative coronal and (**B**) axial CT scan, showing an osseous defect of the petrous bone. The GSPN canal is destructed / widened. Notice the immediate vicinity of the semicircular canals and the involvement of the mastoid cells

**Fig. 8:**
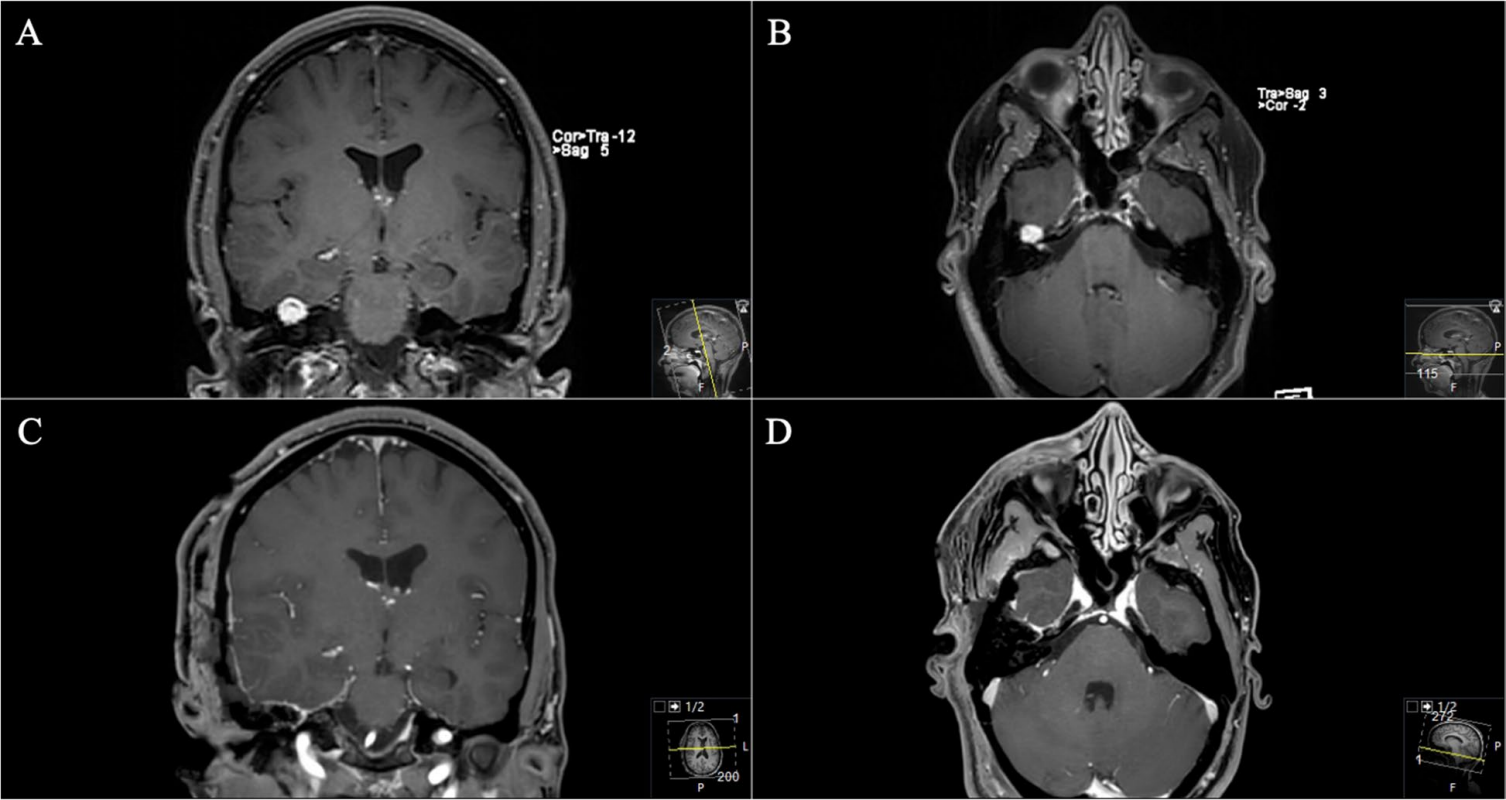
**A** Preoperative coronal and (**B**) axial T1-weighted gadolinium-enhanced MRI, showing the homogenous enhancing schwannoma located at the top of the petrous bone in the middle cranial fossa originating from the GSPN. Notice the accompanying reactive enhancement of the facial nerve in the internal auditory canal **B**. **C** Postoperative coronal and D axial sequences indicating successful complete removal

A 35-year-old woman presented with slowly progressive and then noticeable facial nerve palsy on the right side (House and Brackman grade III) with right-sided deafness for 6 months (Figs. 7 and 8).

#### Surgical approach

An intra-/extradural subtemporal approach was performed. A preauricular straight linear incision and a temporal craniotomy was performed, no further drilling as the small craniotomy was placed at the level of the root of the zygomatic arch. The middle cranial fossa was exposed extradurally, a dural incision was performed to release CSF, so a brain retractor was not necessary anymore. MMA and GSPN exploration were not needed as the tumor could be identified embedded in the petrous bone, covered by a shimmering capsule. was performed for better exposure but was not essential in this case. The capsule was incised, opened and the tumor dissected and removed. Dissection was quite difficult as the facial nerve and the GSPN were impressively thinned out. Tumor volume was 0.8cm^3^. Neuromonitoring with continuous facial EMG was mandatory in this case and was stable throughout the surgery.

#### Histological findings

Portions of a moderately cell-rich schwann-cell tumor were found. Characteristic Antoni-A areas with Verocay bodies were detected. In addition, smaller regressively altered Antoni B areas were present. The tumor cells had no evidence of increased mitotic activity. The examination results led to the diagnosis of a schwannoma, WHO-grade I.

#### Surgical outcome and follow-up

The postoperative course was uneventful. Postoperative MRI confirmed complete removal (Fig. 8). Facial nerve palsy and hearing loss remained unchained.

### Case four

**Fig. 9:**
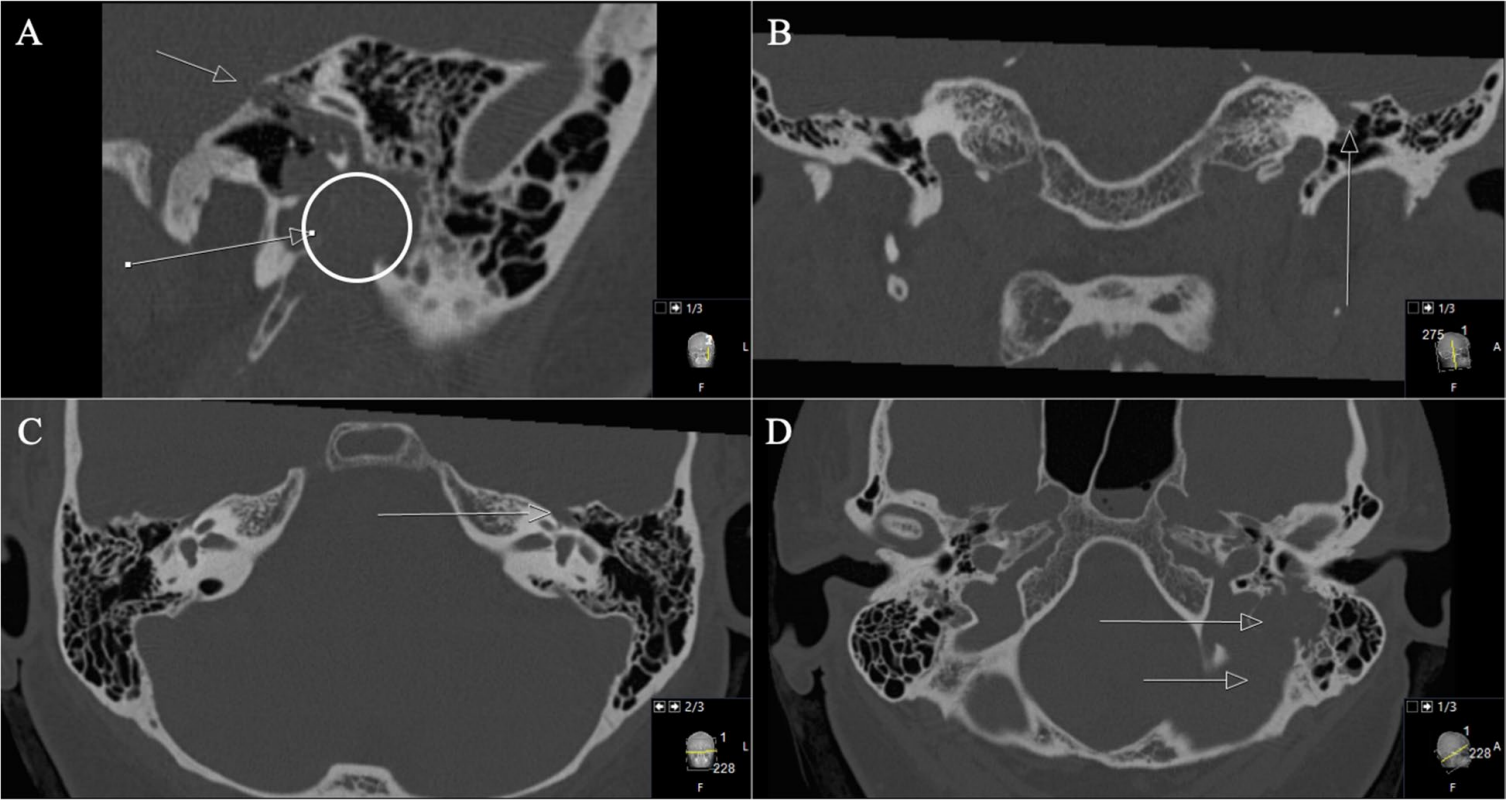
**A**, **B** Preoperative coronal CT scan, demonstrating the infiltrative and destructive behavior of the known GSPN schwannoma (arrowed circle), mastoid cells are shaded and displaced and a widened canal of the facial knee (arrows in **A**, **B**). **C**, **D** Axial sequences, highlighting the widened canal (arrow in **C**) and the osseous destruction of the mastoid and further parts of the petrous bone with the involvement of the jugular bulb and foramen and the sigmoid sinus (arrows in **D**)

**Fig. 10:**
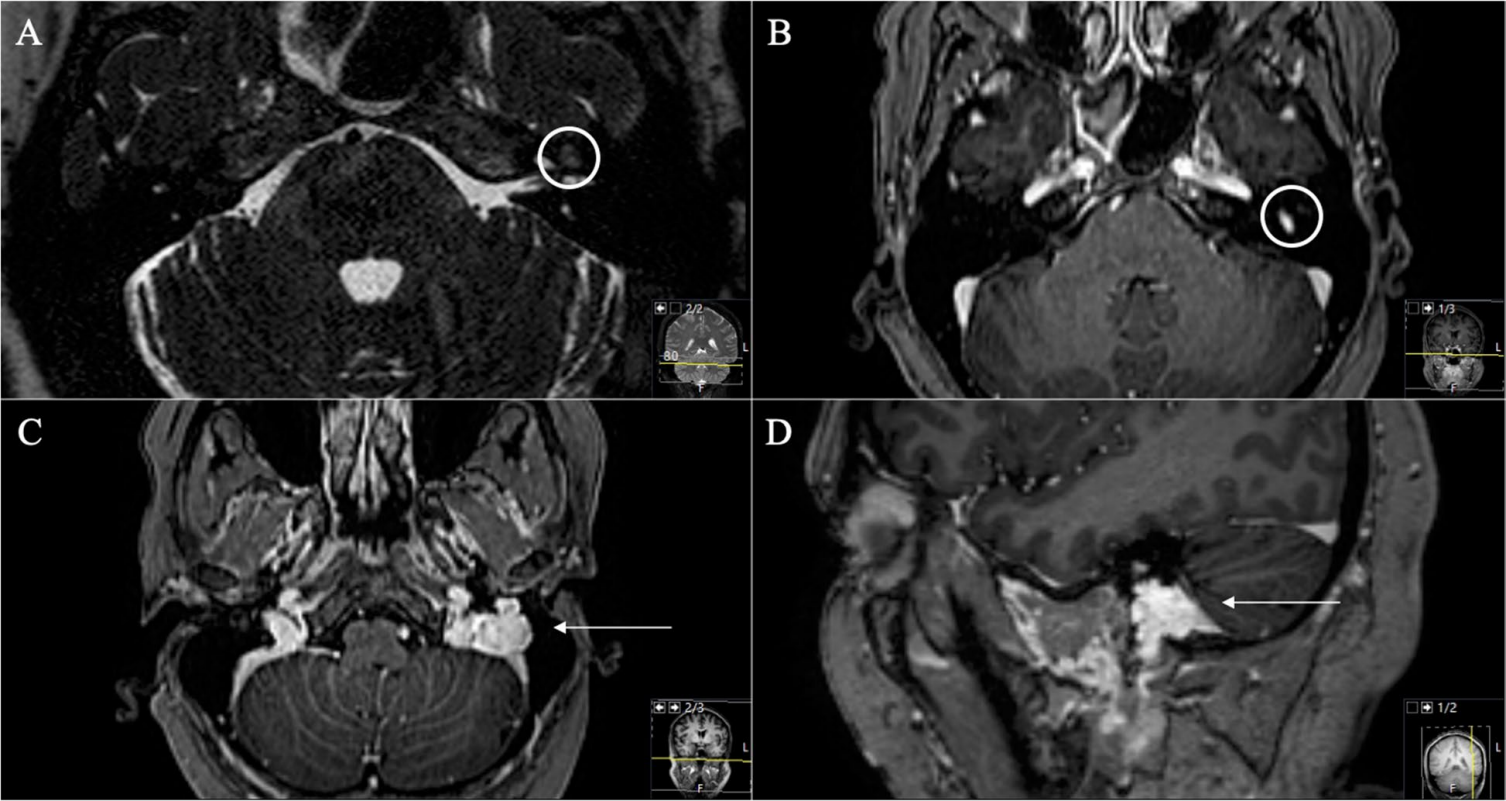
**A** Preoperative axial CISS MRI sequence, highlighting the small hyperintense lesion, which can be easily overseen; remnants of the caudally infiltrating GSPN schwannoma. Following the course of the facial nerve from the knee (**B**), axial T1-weighted gadolinium-enhanced MRI sequences show the enhancement down to the maximal tumor expansion (**C**, **D**) in the petrous bone. The schwannoma remains extradurally with immediate involvement of the jugular bulb and the sigmoid sinus

A 42-year-old woman presented with severe facial nerve palsy. A GSPN schwannoma had already been diagnosed due to facial pain. First, gamma knife radiation was performed. Already at this time, a mild facial nerve palsy on the left side occurred. In the further course, surgery was performed due to massive tumor progression into the petrous bone with the subtotal invasion of the sigmoid sinus and the jugular bulb. Postoperatively a complete facial nerve palsy on the left side (House and Brackman grade VI) occurred. Unfortunately, no initial preoperative images were available at the time of presentation (Figs. 9 and 10). The detected tumor volume was 3.2cm^3^.

#### Surgical approach

**Fig. 11:**
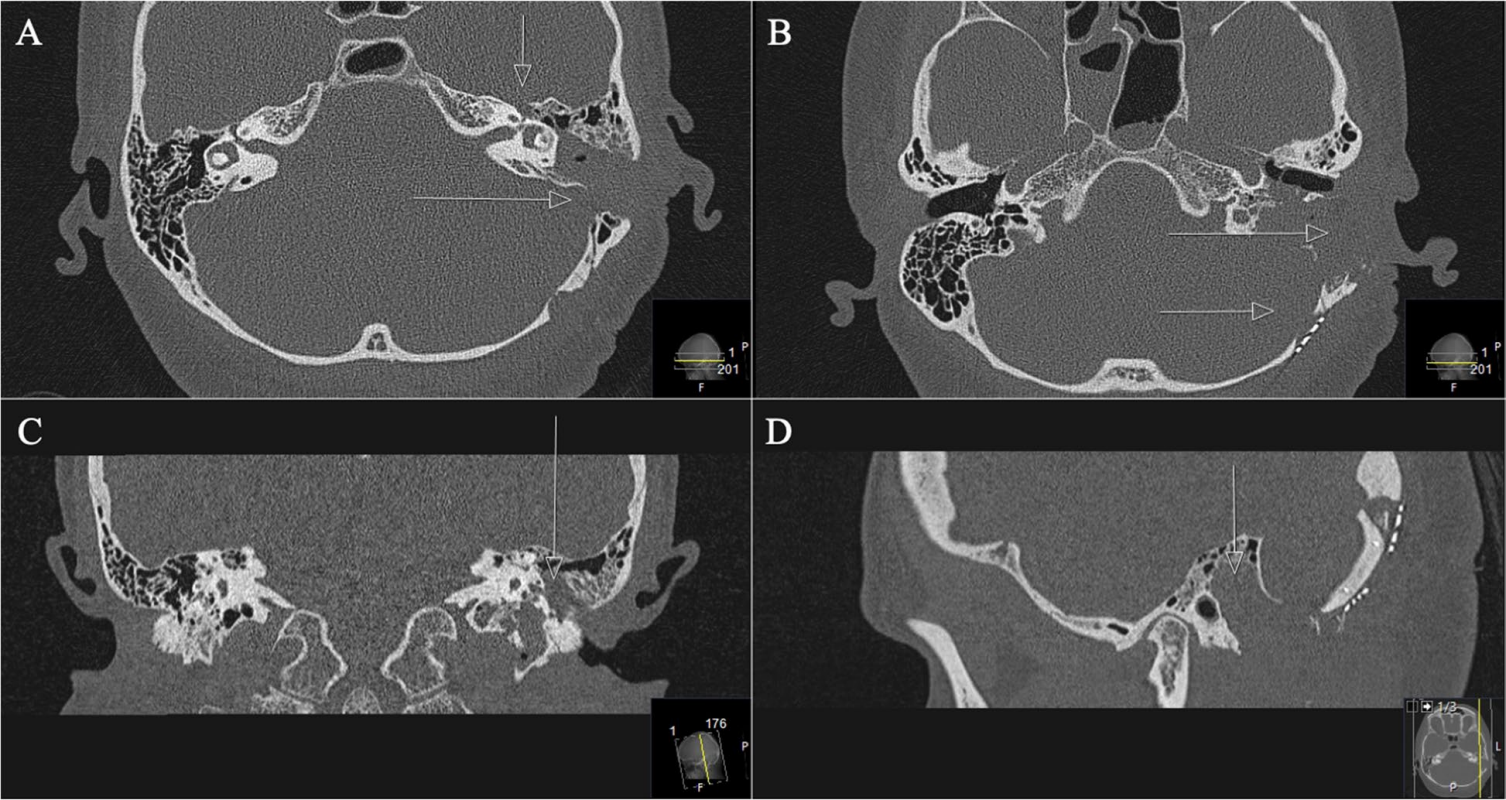
**A**, **B** Postoperative axial, **C **coronal, and **D** sagittal CT scan, displaying the above-mentioned successful mastoidectomy (arrows in **A**, **B**, **D**)and the exposure of the GSPN (arrow in A) and the facial nerve canal (arrow in **C**) throughout meticulous navigated drilling

This case fell out of line as the patient underwent prior gamma-knife treatment and had already undergone surgery via a retrosigmoid approach by ENT colleagues before. According to the surgical reports (initial images were not available), the GSPN schwannoma had an extraordinary extradural invasion into the petrous bone next to the jugular bulb, yet the subtemporal petrous part of the schwannoma was untouched in the postoperative imaging. The existing retroauricular C-shaped incision was extended to a double-C incision in order to expose the mastoid and the presigmoid area. The pre-existing craniotomy was extended, with the superior and anterior margins bordering the transverse and sigmoid sinuses, respectively. A mastoidectomy was carried out to enable a pre- and retrosigmoid corridor. Diffuse bleeding from diploe veins draining into the sigmoid sinus and the jugular bulb was managed with fibrin sealant patch. We remained extradurally and entered the area of the mastoid under continuous drilling. The semicircular canals were used as important landmarks for the further procedure. The capsuled and very adherent tumor was exposed and dissected under difficult circumstances as no facial nerve could be detected at all, and intraoperative stimulation did not show response in the continuous EMG. Decision was made to radically resect the tumor and to sacrifice any facial nerve remnants. Navigated drilling with 2 mm diamond high-speed drill was continued up to the facial knee, further encapsuled tumor was detached from thinned out and pathologically changed GSPN (Fig. 11). The osseous defects were filled with fibrin sealant patch and muscle seals to prevent CSF fistula.

#### Histological findings

Spindle-cell tissue with bland, uniform, pointed cell nuclei was found. Single nuclei were clearly enlarged without mitosis. Immunohistochemically, the reaction against S-100 was positive and MIB1 was positive in two percent of the cell nuclei as well. The examinations led to the diagnosis of a schwannoma, WHO-grade I.

#### Surgical outcome and follow-up

**Fig. 12:**
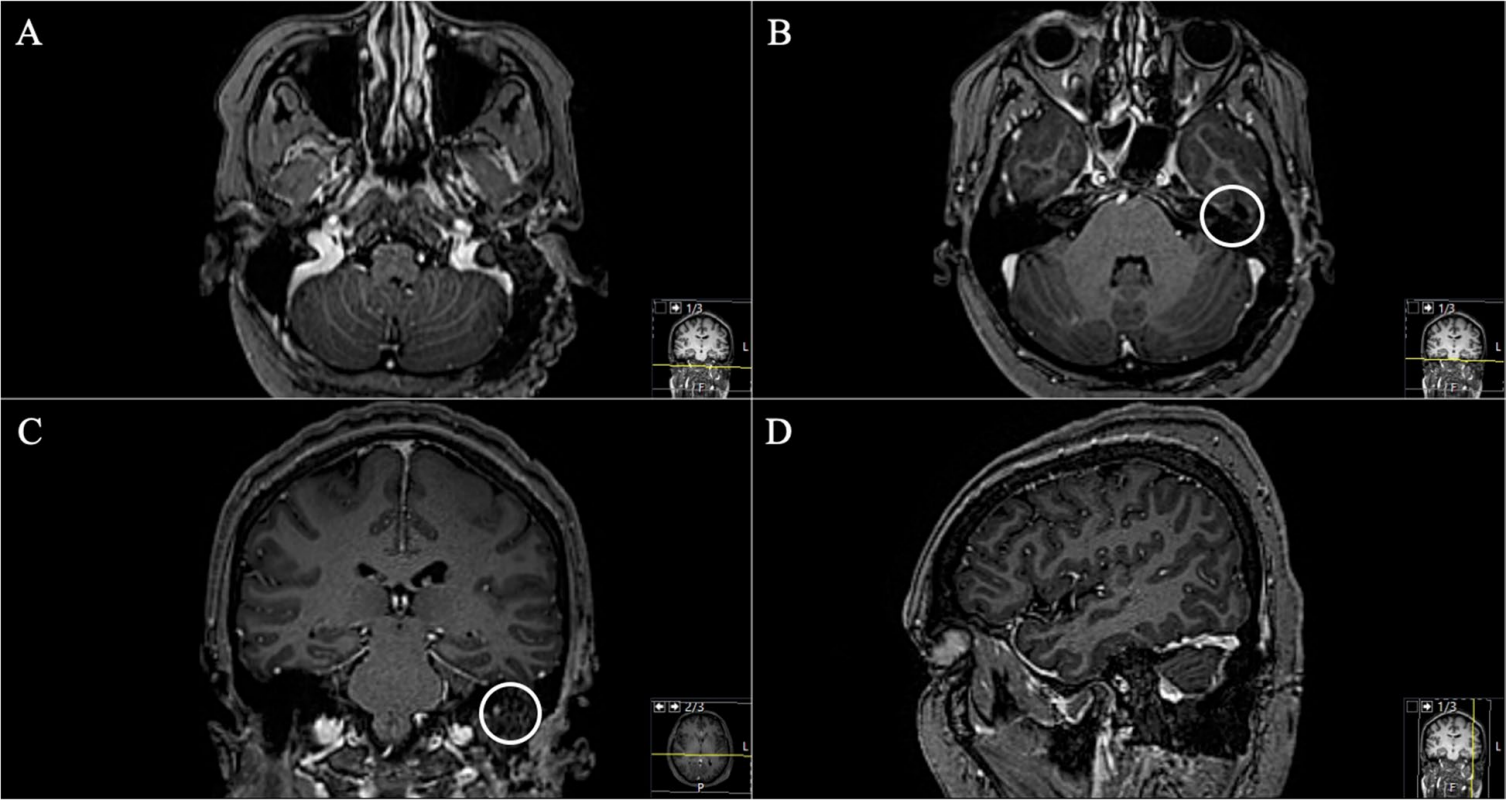
**A**, **B** Postoperative axial, **C** coronal, and **D** sagittal axial T1-weighted gadolinium-enhanced MRI sequences demonstrating successful complete removal (circle)

The subsequent postoperative course was uneventful without new deficits. Postoperative MRI confirmed complete removal (Fig. 12). In the further course, the patient developed a new hearing loss on the left side. Audiometric examination revealed an acute internal ear deafness. High-dose cortisone therapy was initiated immediately as well as antibiotic ear drops. After one week, the patient reported about oto- and rhinoliquorrhea for about three days. A CT scan (Fig. 11) showed regular postoperative findings. However, a CSF leak could not be definitively ruled out. Based on the patient’s symptoms surgical revision was indicated. Intraoperatively, no obvious CSF leak or fistula could be identified, a skull base reconstruction with fascia lata was performed. Postoperatively, no CSF leak occurred.

## Discussion and conclusions

Schwannomas originating from the GSPN are a rare entity, with just 33 cases published in the literature to date (Table 1) [1, 2, 4, 6–22].

**Table 1:** GSPN schwannoma reports and findings. *CN* Cranial nerve, *F* Female, *FU* follow-up, *IQR* Interquartile range, *y* Year, *M* Male, *MFA* Middle fossa approach, *mo* Months, *NA* not available

**Study**	**Nr. of cases**	**Age / Sex**	**Clinical presentation**		**Surgical approach**		**Outcome and FU**	
Tremble and Penfield [6]	1	42/M	CN VII palsy hearing loss		mastoidectomy		FU: NA	
Kleinsasser and Friedmann [7]	1	19/F	CN VII palsy hearing loss		extradural MFA		FU: 6 mo no changes	
Furlow [8]	2	48/M 44/M	seizures CN VII palsy hearing loss		extradural MFA mastoidectomy		FU: 8 mo no changes	
Dolenc and Korsic [9]	1	37/M	CN VII palsy hearing loss		extradural MFA		FU: 1 y no changes	
Kumon et al. [10]	1	21/F	xerophthalmia CN VII palsy hearing loss		extradural MFA		FU: 1y CN VII palsy improvement hearing loss improvement	
Michel et al. [11]	1	20/F	vertigo CN VII palsy hearing loss		extradural MFA		FU: NA CN VII palsy improvement	
Kinouchi et al. [12]	2	58/F 49/F	facial pain vertigo		extradural MFA		FU: NA xerophthalmia	
Aihara et al. [13]	1	65/M	CN VII palsy		extradural MFA		FU: 6 mo CN VII palsy improvement hearing loss improvement xerophthalmia	
Schmidinger and Deinsberger [4]	1	65/F	CN VII palsy hearing loss		extradural MFA		FU: 6 mo hearing unchanged	
Mori et al. [14]	1	50/M	hearing loss		extradural MFA		FU: NA no deficit	
Sade and Lee [15]	1	63/F	CN VII palsy hearing loss		extradural MFA		FU: NA CN VII palsy improvement hearing loss improvement	
Ayberk et al. [16]	1	16/F	diplopia xerophthalmia		extradural MFA		FU: 1 y no deficits	
Amirjamshidi et al. [2]	5	22/F 25/M 28/M 28/M 54/F	case 1: seizures case 2: eye pain case 3: eye irritation, headache case 4: eye pain case 5: hysteria, headache		extradural MFA		FU: NA xerophthalmia in two cases	
Ichimura et al. [17]	4	25/F 27/M 35/F 49/F	case 1: xerophthalmia case 2: seizures, CN VII palsy Case 3: xerophthalmia, CN VII palsy case 4: xerophthalmia, hearing loss		extra-/intradural MFA		FU: NA CN VII palsy with xerophthalmia in two cases CN VII palsy in one case no new deficits in one case	
De Paulis et al. [18]	1	23/F	CN VII palsy hearing loss xerophthalmia		extra-/intradural MFA		FU: NA xerophthalmia	
Umredkar et al. [19]	2	51/F	CN VII palsy hearing loss		extradural MFA		FU: 1 y hearing loss improvement CN VII palsy improvement	
Uppar et al. [1]	1	39/M	headache xerophthalmia		extradural MFA		FU: 2 y CN VII palsy	
Kusumi et al. [20]	1	66/F	vertigo		extra-/intradural MFA		FU: NA no deficits	
Ishikawa et al. [21]	1	69/F	gait disturbance		extra-/intradural MFA		FU: NA no deficits	
Aftahy et al. (present report)	4	50/F 37/M 35/F 42/F	case 1: CN VII palsy, hearing loss case 2: CN VII palsy case 3: CN VII palsy, hearing loss case 4: CN VII palsy, hearing loss		extradural MFA extra-/intradural MFA mastoidectomy pre-/retrosigmoid approach		FU: 9 mo CN VII palsy improvement in one case hearing loss in one case	
**Total in** **n (%) or median [IQR]**	33	38y [25;50,5] 20 F (60.6%) 13 M (39.4%)	CN VII palsy facial pain hearing loss seizures xerophthalmia vertigo diplopia seizures eye pain eye irritation headache gait disturbance	17 (51.5%) 1 (3.0%) 15 (45.5%) 3 (9.1%) 7 (21.2%) 3 (9.1%) 1 (3.0%) 3 (9.1%) 2 (6.1%) 1 (3.0%) 3 (9.1%) 1 (3.0%)	extradural MFA extra-/intradural MFA pre-/retrosigmoid approach mastoidectomy	15 (45.5%) 5 (15.2%) 1 (3.0%) 3 (9.1%)	median FU CN VII palsy hearing loss xerophthalmia Improvement CN VII palsy Improvement hearing loss	9 mo [6;12] 4 (12.1%) 1 (3.0%) 7 (21.2%) 6 (18.2%) 4 (12.1%)

Anatomical knowledge is of utmost importance for correct intraoperative identification as the GSPN schwannomas can originate from any segment of the nerve within the petrous bone [23].

The facial nerve consists of the motor facial nerve and the intermediate nerve including secretory-parasympathetic and gustatory fibers. In the geniculate ganglion, they divide into the facial nerve and the GSPN. The GSPN has an intrapetrosal segment beginning from the geniculate ganglion to the fallopian hiatus. Then, it runs extradurally in its sulcus along the anterior aspect of the petrous bone and perforates the fibrous cartilage of the foramen lacerum, this is defined as the suprapetrosal segment. There, it joins the sympathetic profound petrous nerve, forming the vidian nerve. From here, the pterygoid segment begins: the vidian nerve runs through the pterygoid canal and reaches the sphenopalatine ganglion in the pterygopalatine fossa. Then it switches to the postganglionic neuron innervating the lacrimal, nasal, and palatine glands [4, 9, 24].

Tumors of the geniculate ganglion produce a bulbous enlargement at the geniculate fossa, whereas GSPN schwannomas cause an osseous erosion of the temporal surface of the petrous bone. This is why a scalloping erosion of the anterior superior surface of the petrous bone on CT scans may be seen as pathognomonic for GSPN schwannomas, whereas trigeminal schwannomas lead to petrous apex erosion, as shown in our cases (e.g., Figs. 7 and 9) [1, 18, 24, 25]. Therefore, a CT scan is mandatory to display the presence and extent of bone destruction. Tumor extension into the tympanic cavity or the carotid canal has also been observed [2, 8, 16].

The GSPN can be reliably detected via MRI, and the origin of the tumor can be determined preoperatively [5, 16, 18]. Contrast-enhanced MR images demonstrate homo- to the heterogeneous enhancement of the extra-axial lesion near the geniculate ganglion.

### Surgical approach and alternative treatment options

A subtemporal approach is the recommended technique for targeting the lesion, technical details have been described in detail previously [26–32]. To decide between intradural, extradural, or combined techniques, symptoms and tumor size are major decision criteria [1, 2, 9, 16]. We mainly opted for a combined technique for more detailed inspection and CSF release to reduce temporal brain retraction.

It is up for debate whether the middle fossa base should be drilled out to facilitate tumor removal [13, 18], we used this technique in all cases without adverse events and felt no relevant difficulty. During drilling, it is extremely important to be aware of the anatomy and the location of the petrous carotid artery and labyrinthine structures [33]. Intraoperative neuro-navigation is a highly useful technology that should be included in clinical practice. Furthermore, during the drilling, irrigation is of utmost importance as induced heat is a not negligible risk factor for cranial nerve injury.

GTR should not be forced at any cost due to the mostly benign nature of the schwannomas if neurovascular structures are at risk. De Paulis et al. described cutting the GSPN that could not be dissected from the lesion, thereby minimizing traction on the geniculate ganglion [18], whereas Kusumi et al. left the tumor capsule on the middle fossa to preserve the GSPN and geniculate ganglion [20]. Avoiding traction or direct injury to the geniculate ganglion and the main trunk of the facial nerve is important to avoid postoperative facial nerve palsy or aggravation.

We performed mastoidectomy only in case of extraordinary osseous tumor infiltration. Other authors also described this approach, which became a niche technique due to higher complication rates as CSF leaks [6, 8]. In our case, mastoidectomy was combined with a posterior fossa approach, as the patient was initially managed for the GSPN schwannoma with gamma-knife therapy and was partially resected due to massive tumor progression and destruction of the petrous bone with the affection of the sigmoid sinus and the jugular bulb; an exceptional tumor behavior. In this case, a retrosigmoid approach was performed, as the patient has already been operated on via this corridor. The retrosigmoid approach has already been described as a workhorse approach regarding the posterior fossa and the cerebellar-pontine angle in the literature and should be considered as an alternative to the middle fossa approach [25, 34–36].

Stereotactic radiosurgery (SRS) as an alternative in the management of facial nerve schwannomas has already been described, but specific literature on the use of SRS in GSPN schwannoma is lacking due to the rarity of the condition and due to short follow-up time [37, 38]. Sheehan et al. reported satisfying results: tumor control was 97% after three years and 90% after five years after radiosurgery, respectively [38]. So, SRS is a discussable alternative in patients not suitable for surgery, but also patients with only slight cranial nerve symptoms (e.g., facial nerve palsy grade II or < 50 dB hearing loss at presentation) as functional outcomes show well-preserved nerve functions [38, 39].

GSPN schwannomas are rare entities presenting a myriad of symptoms. Even though they only make up a small fraction of facial nerve schwannomas, they must not be neglected as a differential diagnosis in extra-axial enhancing lesions located in the anterosuperior aspect of the petrous bone. Detailed clinical evaluation and image studies lead to a relatively safe diagnosis and thus facilitate preoperative surgical planning to preserve facial and acoustic nerve functions. Satisfying surgical outcome and also complete remission is possible by GTR.

### Study limitations

Though this was a retrospective case series, we tried to implement a detailed clinical examination and a standardized follow-up protocol based on a certified neuro-oncological board in our clinical workflow. Given the rarity of these lesions, prospective inclusion and follow-up are hard to achieve within a reasonable period. Having this in mind, even though we report a relatively large single-center series, the absolute number of cases does not allow for proper statistical analysis. We recommend that multi-center studies should be conducted to address this problem. Another problem in rare surgical entities is reflected by the changing therapy modalities, which may bias the therapy outcome, the learning curve of the treating surgeons, multiple surgeons involved in the treatment, or changes in the surgical technique.

## Data Availability

The datasets used and/or analyzed during the current study are available from the corresponding author upon reasonable request.

## Abbreviations

CSF: Cerebrospinal fluid
dB: Decibels
EMG: Electromyography
ENT: Ear, nose, and throat
GTR: Gross total resection
GSPN: Greater superficial petrosal nerve
Hz: Hertz
KPSS: Karnofsky Performance Status Scale
MMA: Middle meningeal artery
MRI: Magnetic resonance imaging
SRS: Stereotactic radiosurgery
STR: Subtotal resection
WHO: World Health Organization
